# Removal of Tetracycline Pollutants by Adsorption and Magnetic Separation Using Reduced Graphene Oxide Decorated with α-Fe_2_O_3_ Nanoparticles

**DOI:** 10.3390/nano9030313

**Published:** 2019-02-26

**Authors:** Adriana Magdalena Huízar-Félix, Celia Aguilar-Flores, Azael Martínez-de-la Cruz, José Manuel Barandiarán, Selene Sepúlveda-Guzmán, Rodolfo Cruz-Silva

**Affiliations:** 1 Facultad de Ingeniería Mecánica y Eléctrica, FIME, Universidad Autónoma de Nuevo León, UANL, Ave. Pedro de Alba s/n, Ciudad Universitaria, C.P. 66455 San Nicolás de los Garza, N.L., Mexico; adriana.mhuizarf@gmail.com (A.M.H.-F.); chemistry.aguilar@gmail.com (C.A.-F.); azael70@gmail.com (A.M.-d.-l.C.); 2 Departamento de Electricidad y Electrónica, Universidad del País Vasco (UPV/EHU), 48940 Leioa, Spain; manub@we.lc.ehu.es; 3 Global Aqua Innovation Center and Institute of Carbon Science and Technology, Shinshu University 4-17-1 Wakasato, Nagano 380-8553, Japan; rcruzsilva.rcen@gmail.com

**Keywords:** antibiotic contaminants, electrostatic interactions, magnetic removal

## Abstract

Nanocomposites of reduced graphene oxide (RGO) with ferromagnetic α-Fe_2_O_3_ nanoparticles have been prepared in-situ by thermal treatment. The structure and morphology of the hybrid material were studied by X-ray photoelectron spectroscopy, Raman, X-ray diffraction, and transmission electron microscopy. The results show a hybrid material highly modified with α-Fe_2_O_3_ nanoparticles distributed on the graphene surface. The adsorption kinetics show the presence of α-Fe_2_O_3_ nanoparticles on the RGO surface, and the amount of remaining functional groups dominated by ionization and dispersion. The adsorption kinetics of this adsorbent was characterized and found to fit the pseudo-second-order model. The α-Fe_2_O_3_ nanoparticles on RGO modify the electrostatic interaction of RGO layers and tetracycline, and adsorption properties decreased in the hybrid material. Adsorption isotherms fit with the Langmuir model very well, and the maximum capacity adsorption was 44.23 mg/g for RGO and 18.47 mg/g for the hybrid material. Magnetic characterization of the hybrid material shows ferromagnetic behavior due to the nanosize of α-Fe_2_O_3_ with a saturation magnetization, Ms = 7.15 Am^2^/kg, a remanence Mr = 2.29 Am^2^/kg, and a coercive field, Hc = 0.02 T.

## 1. Introduction

Antibiotics have become an important water pollutant owing their intense use over the past decades. Since their introduction in 1950, tetracyclines are one of the major types of antibiotics, besides sulfonamides, macrolides, and quinolones that have been extensively used in human and veterinary medicines and as growth promoters in agriculture and aquaculture [1]. The third generation tetracyclines were approved by the Food and Drug Administration in 2005 and after a decade of clinical use, remain relatively active against many multidrug resistance pathogens including *Staphylococcus aureus, Acinetobacter baumannii, Klebsiella pneumoniae,* and *Escherichia coli* [2]. There are many pathways for the environmental release of antibiotics, but in general, any anthropogenic activity which demands its use has exposed it to the contamination of soils and sediments and passed it to subsurface water, lakes, and rivers [3]. Studies have warned about the potential ecological risks and public health threat due to the increased antibiotic resistance of gens and bacteria that leads to a bigger issue since they cannot be easily eliminated [4]. The occurrence of tetracyclines in surface water and soils is becoming a worldwide issue, and the toxicity effect eventually will reach the ecosystems [3,5]. The more significant techniques to remove micropollutants from contaminated media include adsorption, biodegradation, photodegradation, and the advanced oxidation process [6]. Some of them face many challenges due to their high operational costs and time-consuming processes. Adsorption is an inexpensive technique easy to implement, with high efficiency, and without toxic by-products. The effectiveness of the adsorption process depends on the physicochemical properties of the adsorbent, such as specific surface area, porosity, surface polarity, and morphology of the material, as well as the characteristics of the adsorbate, such as size, polarity, ionic charge, and hydrophobicity. The adsorption mechanisms consist of the electrostatic interaction and physical bindings of adsorbate to the specific sites on the surface of an adsorbent [7,8]. Activated carbons are frequently used as adsorbent owing to the high specific surface area, pore size, and chemical functionalities [9]. In recent years, nanocarbons, including carbon nanotubes (CNT) and graphene-like materials, have been widely applied as adsorbents for several organic contaminants including antibiotics [8]. Graphene consists of graphitic carbon sheets with a hexagonal structure of carbon atoms linked together by sp^2^ bonds and which exhibit excellent mechanical and physical properties [10]. Graphene can be obtained by bottom-up methods, such as epitaxial growth and chemical vapor deposition. However, top-down approaches, such as the mechanical and chemical exfoliation, are the most convenient methods for large production of graphene-type materials [11]. Graphene oxide (GO) results from the chemical oxidation of graphite and subsequent exfoliation, leading in a modified graphene with a large number of oxygen functional groups (carboxyl, hydroxyl, carbonyl, and epoxy) adding sp^3^ domains to the structure [12]. GO can be reduced to remove oxygen content restoring the conjugated structure and increasing the electrical properties [13]. Graphene-based materials are promising candidates as adsorbents due to good chemical stability, large surface area, and the remaining functional groups as well the domains of conjugated π structure which allow the strong interaction between the surface of graphene and pollutants. Recently, the adsorption of tetracycline on nanocarbons materials have been reported (Table 1). Pristine and modified CNT have been used for the removal of tetracycline through adsorptive interactions, such as π–π electron–donor–acceptor interactions and cation–π bonding [14,15]. However, the most graphene-based material used for adsorption of tetracycline (TC) is GO and GO composites trough chemisorption via π–π interaction and cation–π bonding and the reported data (Table 1) also show that the addition of a second phase impact on the adsorption properties, such as *Q_max_* and adsorption conditions (pH and temperature) [16,17]. Macroporous polystyrene/GO composites showed a high adsorption capability of tetracycline at pH 6 and resulted in an effective recyclable adsorbent [18]. In addition, GO modified with metal oxide or metal nanoparticles have been studied as adsorbents for several antibiotics and in some cases, exhibit a positive effect, however, some of them reduce the specific area and adsorption sites. Nevertheless, modified GO is desirable for the presence of functionalities that in turn can improve the removal system promoting degradation and ease separation from the solution [19,20,21]. The photocatalytic degradation of TC has been reported using GO modified with TiO_2_/Fe_2_O_3_ nanoparticles as a magnetic adsorbent and catalyst [22]. The magnetic separation is a simple method based on the selective recovery of the magnetic adsorbents using a magnetic field and has been reported for several carbonaceous materials [23,24]. Magnetic composites of GO with paramagnetic electrolytes were prepared by electrostatic interactions and then used as adsorbent for TC removal [25]. In addition, the adsorption of TC and heavy metal ions on reduced graphene oxide (RGO) with different oxidation degrees and modified with Fe_3_O_4_ nanoparticles has been recently reported [26]. The reports show that functionalities and adsorption properties of reduced graphene oxide are modified by the preparation method that impacts their surface chemistry due to the presence of oxygen and nitrogen functionalities. RGO exhibits a low number of oxygen functional groups to provide an electric charge in aqueous media. However, adding additional nanostructures might expand the surface chemistry and physical properties to generate interesting adsorbents. Few works have been focused on the study of the structure of modified RGO and the adsorption properties of organic materials. In this work, the in situ synthesis of a hybrid material of reduced graphene oxide and α-Fe_2_O_3_ nanoparticles is presented, and the structure and morphology were studied by spectroscopy and electron microscopy techniques. In addition, the adsorption properties of the hybrid materials were studied as a function of pH using tetracycline in aqueous medium. The magnetic properties of the nanocomposite were also evaluated to study the potential as magnetic removal adsorbent.

## 2. Materials and Methods

### 2.1. Materials

Graphite (natural flakes), tetracycline hydrochloride (CAS 64-75-5), and FeCl_3_∙6H_2_O (ACS reagent, 97%) were purchased from Sigma-Aldrich (Toluca, Mexico). GO was obtained by oxidizing graphite through Marcano’s method [12]. Other chemicals used to prepare buffer solutions were of analytical reagent grade and used without further purification. Deionized water was used in all experiments.

### 2.2. Synthesis of RGO and α-Fe_2_O_3_/RGO Hybrid Materials

**Figure 1:**
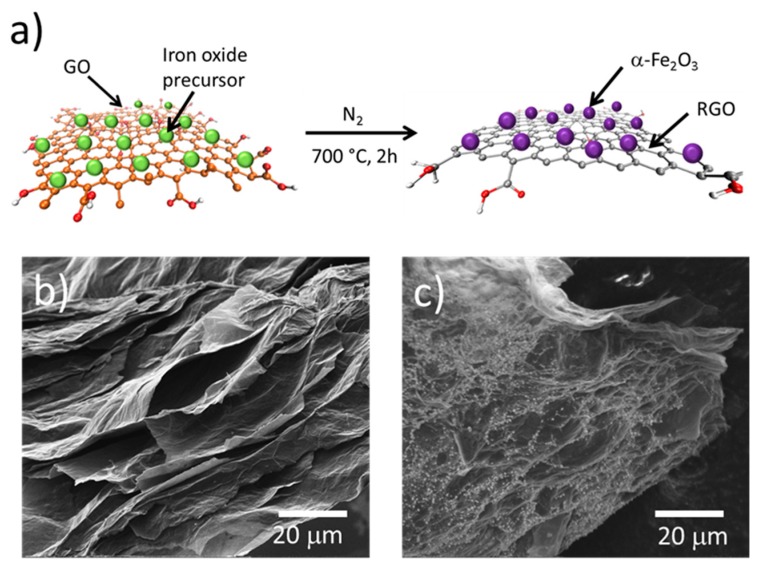
(**a**) Schematic of production of α-Fe_2_O_3_/RGO hybrid material by the simultaneous thermal reduction of graphene oxide (GO) and synthesis of iron oxide nanoparticles; (**b**) Scanning electron microscopy (SEM) image of RGO prepared by the thermal reduction at 700 °C as control and (**c**) SEM image of the resulting α-Fe_2_O_3_/RGO hybrid material.

A GO paper was obtained by casting of a GO dispersion (3 mg/mL) in a petri-dish and dried at room temperature. RGO resulted from the thermal treatment of a GO paper at 700 °C for 2 h using a heating rate of 5 °C/min under N_2_ flow. The modified RGO decorated with iron oxide nanoparticles was obtained by the simultaneous thermal reduction of GO paper loaded with FeCl_3_∙6H_2_O as an iron oxide precursor. A mass ratio of 1:1 of RGO:Fe was aimed for considering about 63% mass loss of GO during the thermal treatment at 700 °C. The GO loaded with FeCl_3_∙6H_2_O was prepared by casting and then thermal treatment at 700 °C for 2 h under N_2_ flow using a heating rate of 5 °C/min. A schematic illustration for the one-step synthesis of α-Fe_2_O_3_/RGO nanocomposites is shown in Figure 1a. After thermal treatment, the material was subjected to three centrifugation/redispersion cycles with deionized water and dried at 40 °C during 24 h.

### 2.3. Adsorption Experiments of Tetracycline on RGO and α-Fe_2_O_3_/RGO Hybrid Materials

Kinetic studies were carried out using a 0.6 mg/mL of adsorbent and a concentration of tetracycline in the range of 30 to 8 mg/L. The kinetics experiments were performed for different pH of the media (4, 7, and 10) and acquiring the absorbance at 357 nm for 90 min. Adsorption isotherms of tetracycline on RGO and α-Fe_2_O_3_/RGO hybrid material were carried out varying the tetracycline concentration (2−25 mg/L) in different pH buffer solutions (4, 7, and 10) using 3 mg of adsorbent in a 7 mL cell. The dispersion was separated by centrifugation and adsorption of the supernatant was measured after 2 h to reach the equilibrium conditions.

### 2.4. Characterization α-Fe_2_O_3_/RGO Hybrid Materials

X-ray diffraction (XRD) analysis for all samples was performed using a Bruker D8 Advance powder diffractometer (Billerica, MA, USA) with Cu radiation. The diffraction patterns were acquired between 5° and 90° of 2θ, with a scan rate of 0.5°/min and an incident wavelength of 0.154 nm (Cu Kα). Fourier-transform infrared spectroscopy (FTIR) measurements were carried out in a Thermo Scientific Nicolet 6700 FTIR spectrometer (Waltham, MA, USA) with a wavelength range of 4000 cm^−1^ to 400 cm^−1^ in transmission mode by using KBr pellets. Scanning electron microscopy (SEM) characterization was performed in an FEI NOVA NANOSEM 200 (Hillsboro, OR, USA) at 10 KV of accelerating voltage and 5 mm of working distance. Transmission electron microscopy (TEM) images were obtained in an FEI Titan G2 80-300 TEM (Hillsboro, OR, USA) with an accelerating voltage of 300 KV. A drop of either RGO or α-Fe_2_O_3_/RGO aqueous dispersions was dried onto a Lacey Silicon film on a copper grid. Raman spectroscopy measurements were done using a Thermo Fisher Scientific DRX Raman Microscope (Waltham, MA, USA) with a laser line of 520 nm in the range of 50 cm^−1^ to 3500 cm^−1^. The samples were placed directly in the sample holder. The chemical surface composition of the samples was studied by X-ray photoelectron spectroscopy (XPS) using a Thermo Scientific K-Alpha- X-ray Photoelectron Spectrometer System (Waltham, MA, USA). The analysis was done with monochromatized Al Kα radiation (E = 1486.68 eV). The analysis was carried out acquiring the survey spectra with a pass energy of 200 eV and the high-resolution core-level spectra for C 1s, O 1s, and Fe 2p using a pass energy of 30 eV under ultra-high vacuum conditions. The adsorption kinetic of tetracycline in RGO and α-Fe_2_O_3_/RGO were monitored in situ using a Cary 5000 UV-Vis-NIR Spectrophotometer. Magnetic measurements were carried out using a Vibrating Sample Magnetometer (VSM developed at the University of the Basque Country, Leioa, Spain) mounted onto a Cryogenics platform for magnetic measurements with a superconducting magnet of 14 T, a vibration frequency of 20 Hz, sensitivity of 10^−6^ emu with a preamplifier of 5 s average.

## 3. Results

### 3.1. Synthesis of α-Fe_2_O_3_/RGO

Thermal treatment of GO has been long known as a method to reduce graphene oxide. It can be carried out in a wide range of temperatures; however, the microstructure of resulting RGO is strongly affected by the reduction conditions. FTIR analysis shows that after reduction at 700 °C, only a small amount of oxygen functional groups which correspond to C=O (carbonyl), C–O (carboxyl), and C–O (epoxide), besides C=C (Figure S1) remains on the RGO. The remaining functionalities, mainly at the edges of the RGO sheets, make possible the electrostatic interaction in addition to the π–π interactions with organic molecules, inorganic materials and atoms/ions [31]. The synthesis of modified RGO sheets by thermal treatment of GO paper loaded with metal oxide precursor was carried out by thermal treatment. The electrostatic interaction between oxygen functional groups of GO and Fe^+3^ ions is well-known, and allows the nucleation and growth of nanoparticles on the RGO surface during the thermal treatment, and the iron oxide nanoparticles result from the reaction between Fe^+3^ ions and the released oxygen from GO during the thermal reduction, as shown in Figure 1a. The SEM image in Figure 1b shows the homogeneous distribution of iron oxide particles on the α-Fe_2_O_3_/RGO composite surface, very different from the RGO sample that shows a surface free of particles (Figure 1b,c). Even though thermal treatment of GO is accompanied by the elimination of H_2_O and CO_2_ that promote exfoliation, there are still thick sheets attributed to restacked RGO sheets.

### 3.2. Microstructural Characterization of α-Fe_2_O_3_/RGO Hybrid Materials

**Figure 2:**
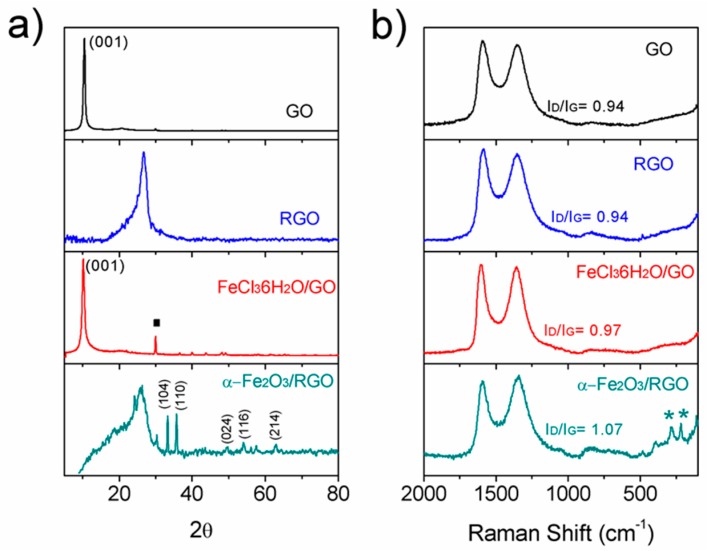
(**a**) X-ray diffraction (XRD) patterns of GO, reduced graphene oxide (RGO), FeCl_3_·6H_2_O/GO, and α-Fe_2_O_3_/RGO hybrid material, planes and crystalline phases are marked; and (**b**) the Raman spectra of GO, RGO, FeCl_3_·6H_2_O/GO, and α-Fe_2_O_3_/RGO hybrid material showing the microstructural characterization.

The GO diffraction pattern exhibits a reflection peak about 10.5° of 2θ associated to the 001 spacing of 0.84 nm of the GO sheets. After thermal reduction, the XRD diffraction pattern shows a broad peak with a maximum at 26.7° corresponding to a turbostratic structure with a mean spacing of 3.4 Å (Figure 2a). For the FeCl_3_·6H_2_O/GO hybrid paper, a shift of the GO 001 peak to a lower diffraction angle, due to an increment in the spacing due to the presence of the iron chloride salt, was observed. The respective diffraction pattern shows small peaks corresponding to the FeCl_3_·6H_2_O used as an iron oxide precursor. After thermal reduction, the XRD analysis shows that the FeCl_3_·6H_2_O/GO nanocomposite paper was reduced to a hybrid material of turbostratic RGO loaded with iron oxide nanoparticles with rombohedrical crystalline structure of α-Fe_2_O_3_ according to the presence of two intense peaks at 33.15° and 35.6°, associated to the (104) and (110) planes respectively (JCPDS—PDF card 330664). The broad signal with a maximum at 25.6° corresponds to a turbostractic plane RGO which is broader than that of the pure RGO due to the presence of nanoparticles between the sheets that result in a more disordered carbonaceous structure.

Figure 2b shows the Raman spectra of GO, RGO, FeCl_3_·6H_2_O/GO, and α-Fe_2_O_3_/RGO hybrid material. The spectra display mainly two intense and broad peaks at 1344 cm^−1^ and 1589 cm^−1^, which correspond to the D band and G band of carbon, respectively. The G band is associated with the first-order scattering of the E2g mode observed for sp^2^ carbon domains, and the D band is associated with structural defects or edges that break the symmetry and selection rule [32]. This suggests that all samples contain highly disordered and graphitic layers. The intensity ratio of D band to G band (ID/IG) is used as a measure of the disorder. The ID/IG ratio for the α-Fe_2_O_3_/RGO hybrid material is higher than that of RGO suggesting that the nanoparticles increase the structural disorder of the hybrid material.

**Figure 3:**
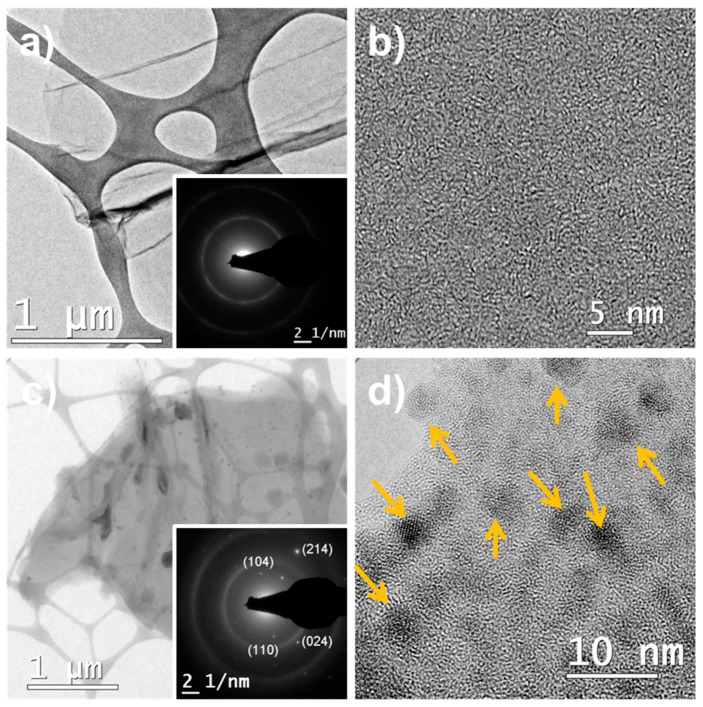
Transmission electron microscopy (TEM) analysis of (**a**,**b**) RGO as control and (**c**,**d**) α-Fe_2_O_3_/RGO hybrid material. Insets in (**a**) and (**c**) show the selected-area electron diffraction (SAED) analysis for the respective materials.

Microstructural characterization was also studied by TEM (Figure 3). The low magnification image of the RGO sample (Figure 3a), used as control, shows a layered structure with a lateral size larger of 1 µm and wrinkled morphology typical of RGO. The absence of crystalline domains in the high-resolution TEM (HRTEM) image of RGO (Figure 3b) and the respective selected area electron diffraction pattern (Figure 3c) showing two rings, confirm the disordered structure resulting from the short-time and low-temperature thermal treatment. The study of α-Fe_2_O_3_/RGO hybrid material by TEM shows the wide particle size distribution of α-Fe_2_O_3_ nanoparticles dispersed on the RGO surface, the particle sizes range from 2 nm to 50 nm. The TEM image at low magnification show RGO layer with agglomerates of α-Fe_2_O_3_ nanoparticles (Figure 3d). The wide particle size distribution is common in composite materials prepared by in situ techniques, but this method is simple and allows large-scale production, and avoids the use of chemical reducing agents. HRTEM image shows smaller crystalline α-Fe_2_O_3_ nanoparticles attached to the RGO sheet showing a large number of nanoparticles consistent with the amount of metal oxide precursor added. The selected-area electron diffraction (SAED) presents diffraction spots associated with (214), (110), (024), and (104) planes in the rhombohedral crystalline structure of α-Fe_2_O_3_ and two diffraction rings associated with the RGO structure.

**Figure 4:**
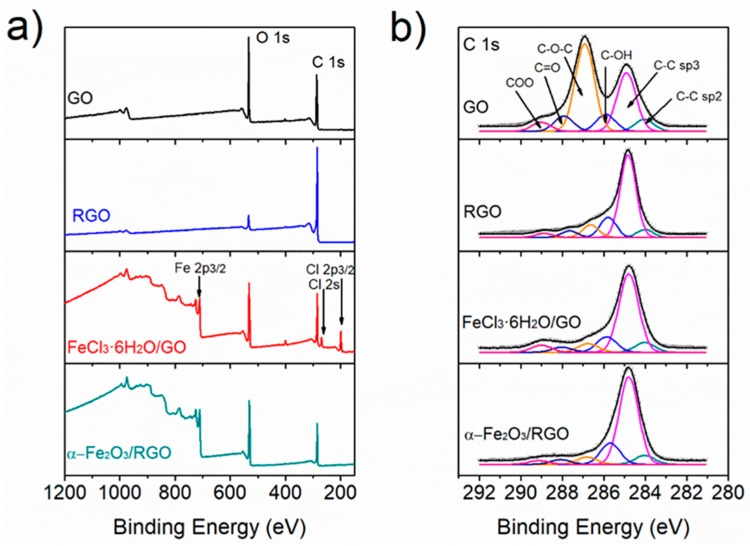
X-ray photoelectron spectroscopy (XPS) analysis of GO, RGO, FeCl_3_·6H_2_O/GO, and α-Fe_2_O_3_/RGO hybrid material. (**a**) Survey spectra and (**b**) spectra of C 1s core.

Surface chemical composition was carried out by XPS analysis (Figure 4). Survey spectra (Figure 4a) shows that the elemental composition of GO and RGO samples consist of C and O. However, RGO spectrum exhibits a lower contribution of oxygen due to the removal after thermal treatment. The spectra for the FeCl_3_·6H_2_O/GO and α-Fe_2_O_3_/RGO samples present additional contribution of Cl and Fe, associated with traces of the precursor and iron oxide nanoparticles. In addition, there is no significant change in the oxygen contribution due to the presence of iron oxide.

The spectra C 1s core for GO, RGO, FeCl_3_·6H_2_O/GO, and α-Fe_2_O_3_/RGO (Figure 4b) were deconvoluted into six components at 284 eV, 284.8 eV, 285.7 eV, 286.8 eV, 288.1 eV, and 289.1 eV assigned to the following electronic environments: carbon atoms C–C (sp^2^ and sp^3^), C–OH, C–O–C, C=O, and COO, respectively [33]. The intensity of each component is associated with its chemical abundance and after the thermal treatment, the relative contribution of C–C components increased, whereas the components associated with oxygen functional groups decreased due to the thermal reduction of GO through elimination of CO, CO_2_, and H_2_O leading to restacking and loss of mass [34]. This can be seen in the relative composition of carbon species in the samples (Table S1). The results show oxygen functionalities in α-Fe_2_O_3_/RGO hybrid material that in turn promote the interaction with other molecules. In addition, despite the fact that no reduction treatment was carried out during the preparation of FeCl_3_·6H_2_O/GO paper, the intensity of C–O–C component in the C 1s spectrum (6.9%) is closer to that in RGO (9.4%) than in the GO spectrum (44%) possibly due to the anchoring of Fe^+3^ ions on GO functionalities. This was further corroborated by the analysis of the O 1s core spectra (Figure S2a) where a contribution of Fe-O is observed in the chemical structure of FeCl_3_·6H_2_O/GO material. The analysis of the Fe 2p core spectra for the FeCl_3_·6H_2_O/GO and α-Fe_2_O_3_/RGO materials shows the presence of Fe^+3^ (Figure S2b). Details of the chemical composition of the materials as well the surface area measurement of the RGO and α-Fe_2_O_3_/RGO via nitrogen gas adsorption yielded a Brunauer, Emmett, and Teller (BET) is presented in the Table S1, and the N_2_ isotherm adsorption curves can be seen in Figure S3.

### 3.3. Tetracycline Adsorption Studies

**Figure 5:**
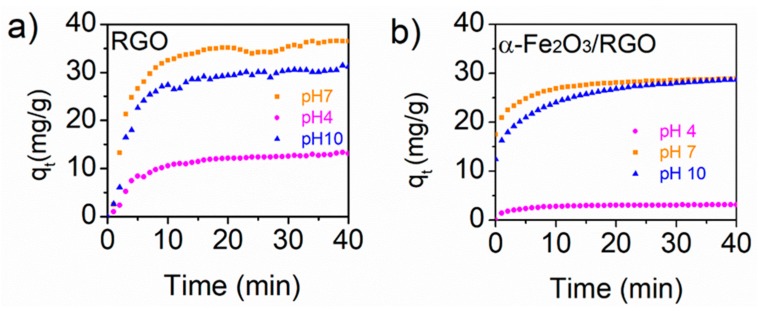
(**a**) Kinetic adsorption of tetracycline on RGO and (**b**) on α-Fe_2_O_3_/RGO hybrid material as a function of pH.

The kinetics of adsorption of tetracycline on RGO as control and α-Fe_2_O_3_/RGO hybrid material were studied under different pH (Figure 5) and under those conditions (pHs 4, 7, and 10), tetracycline hydrochloride solution is stable, and no precipitation was observed. The kinetics curves, plotting the amount of tetracycline adsorbed (q_t_, mg/g) in time, show that equilibrium is reached after 30 min showing maximum adsorption at pH 7 for the α-Fe_2_O_3_/RGO and RGO system. The adsorption kinetics was modeled using the pseudo-second-order model the parameters for each system are presented in Table S2. The pseudo-second-order model was chosen due to its wide use for the adsorption of organic pollutants on graphene-based adsorbents and because it assumes that the rate-limiting step involves chemisorption [16]. Several reports show that the charge of TC depends on pH value and can exist as a cationic, zwitterionic, and anionic species under acidic, moderately acidic to neutral, and alkaline conditions [35]. However, the capability of adsorption of TC on RGO depends also on the specific surface area, and the amount of remaining carboxylic groups that provide ionization groups in RGO and both features can be tuned by the reduction and activation process. In this case, since no additional activation was carried out, tetracycline adsorption could be driven by π–π interactions and cation–π bonding. Results show that the amount of remaining functional groups in RGO leads to an adsorption of 38.55 mg/g, 32.88 mg/g, and 14.58 mg/g of TC at pH 7, 10, and 4, respectively, but decreased at 29.33 mg/g, 29.85 mg/g, and 3.21 mg/g at pH 7, 10, and 4, respectively, when α-Fe_2_O_3_/RGO was used as adsorbent under these experimental conditions; 25 °C and 0.6 mg/ml of adsorbent and initial tetracycline concentration of 8.25 mg/L, 10.09 mg/L, 12.66 mg/L for pH 10, pH 7, and pH 4 respectively, since the kinetics parameters are affected by the initial concentration of tetracycline and the concertation ratio of adsorbent and adsorbate [16]. A higher initial concentration might increase competition between TC molecules for given adsorption sites on α-Fe_2_O_3_/RGO accordingly a lower K_2_ value was obtained (Table S2). The decrease in adsorption compared with RGO material could be attributed to the decreasing of oxygen functional groups, and the presence of α-Fe_2_O_3_ nanoparticles on RGO active sites. Thermal reduction of GO results in loss of oxygen functional groups that increased the positive charge of the molecule, while the presence of α-Fe_2_O_3_ hindered the electrostatic interaction between the adsorbent and the tetracycline and the removal efficiency of the tetracycline decreased on α-Fe_2_O_3_/RGO material.

Thermodynamics of the adsorption studies was carried out obtaining isotherms as a function of pH of the media. The concentration of tetracycline varied from 25 to 5 mg/L and the amount of the tetracycline adsorbed on the α-Fe_2_O_3_/RGO hybrid material and the remaining tetracycline in the solution were recorded after 2 h. The resulting adsorption data were fitted with the Langmuir model which relates the amount of adsorbate adsorbed per unit weight of adsorbent (*q_e_*, mg/g) and the concentrations of adsorbate in the bulk solution (*C_e_*, mg/L) at a given temperature under equilibrium conditions (Table S2). The Langmuir model [36] assumes uniform energies of adsorption on the surface and no transmigration of adsorbate in the plane of the surface. It is linearly expressed as:

(1) $$\frac{C_{e}}{q_{e}}=\frac{1}{Q_{max}K_{L}}+\frac{C_{e}}{Q_{max}}$$

where *Q_max_* y *K_L_* are Langmuir constants related to the maximum adsorption capacity (mg/g) and the adsorption energy (L/mg), respectively. Adsorption data were also fitted with the Freundlich model [37], that assumes heterogeneous adsorption and multilayers. It can be expressed linearly as:

(2) $$\log q_{e}=\log K_{f}+\frac{1}{n}\log C_{e}$$

where *k_F_* is the constant of Freundlich and n is the heterogeneity factor associated with the adsorption capacity.

**Figure 6:**
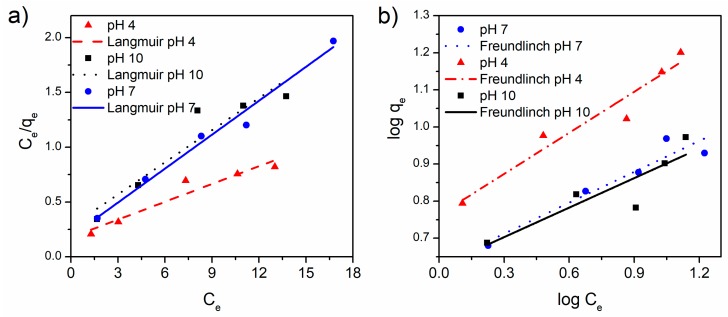
(**a**) Langmuir and (**b**) Freundlich model fitting for the adsorption of tetracycline on α-Fe_2_O_3_/RGO hybrid material. Experiment condition: 25 °C; 0.7 mg/mL of adsorbent.

The results indicated that the adsorption data fitted reasonably with Langmuir and Freundlich isotherms (Figure 6). However, RGO isotherms fitted very well with the Freundlich model whereas the adsorption isotherm for α-Fe_2_O_3_/RGO fits better with the Langmuir model. Table 2 presents the kinetics constants associated with the isotherms evaluated at different pH values. The value of n constant for RGO isotherms range between one to two, which indicates heterogeneity in the adsorption capacity owing to the edges, multilayer structure for restacking, and different sp^2^ and sp^3^ domains in RGO. The n constant values for α-Fe_2_O_3_/RGO isotherms fitted with the Freundlich model were larger than two for all the analyzed pH values, which indicates that this adsorbent exhibit a surface heterogeneity owing the presence of two phases, RGO and α-Fe_2_O_3_ nanoparticles each one with different surface chemistry. Then the results show the disturbance of the adsorption of tetracycline by the presence of different surface chemistries in the adsorbent layer which resulted in low adsorption efficiency. This agrees with the constants obtained from the fitting with the Langmuir model, the maximum adsorption (*Q_max_*, mg/g) of RGO isotherms were 44.23 mg/g, 39.94 mg/g, and 15.82 mg/g at pH 7, 10, and 4, respectively. The *Q_max_* for the α-Fe_2_O_3_/RGO decreased to 9.69 mg/g, 10.25 mg/g, and 18.48 mg/g at pH 7, 10, and 4, respectively, the decreasing is related to the modified functionalities in RGO surface by adding nanoparticles with wide particle size hindering the active sites of RGO. The maximum adsorption of tetracycline on α-Fe_2_O_3_/RGO was observed under pH 4 and agrees with previous reports for chemisorbing. The adsorption results are lower than those reported for GO in the literature (Table 1). Unlike RGO, GO has a higher abundance of functional groups, such as carboxylic, phenolic, hydroxyl, and epoxy groups. These groups provide GO with water dispersibility and stabilize the carboxylate group favored the electric charge needed for the chemisorption of tetracycline. In addition, the results also show that α-Fe_2_O_3_ nanoparticles on RGO hindered tetracycline adsorption. Additional studies can be performed to improve the adsorption capability by activating the surface and to find the optimum conditions to tetracycline adsorption by modifying adsorption conditions, such as ion strength.

### 3.4. Magnetic Characterization of α-Fe_2_O_3_/RGO Nanocomposites

**Figure 7:**
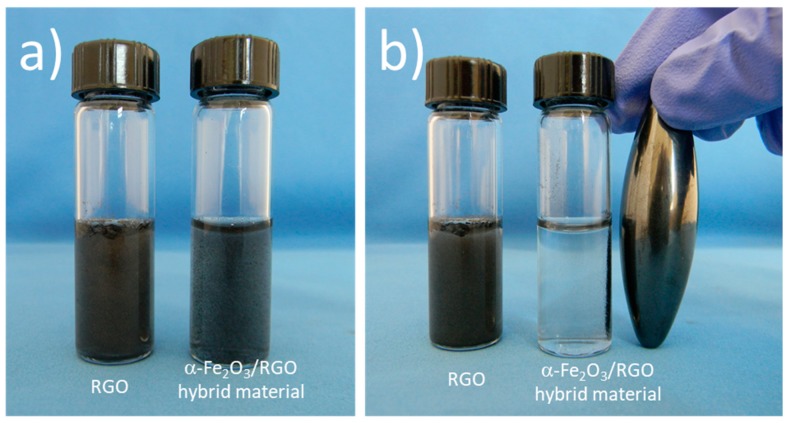
Pictures of RGO and α-Fe_2_O_3_/RGO hybrid material dispersion in pH 4 buffer solution (**a**) before and (**b**) after exposing a magnetic field.

Figure S5 shows the measurement of the temperature dependence of magnetization of the α-Fe_2_O_3_/RGO nanocomposites (Figure S5a) and the ferromagnetic character of the α-Fe_2_O_3_ nanoparticles with a Curie temperature of 567 °C. The magnetic hysteresis curves were recorded at 300 K (room temperature). Figure S5b indicates a saturation magnetization Ms = 7.15 Am^2^/kg, a remanence of Mr = 2.29 Am^2^/kg, and a coercive field of Hc = 0.02 T. The magnetic intensities are low due to the presence of RGO and the small size of the α-Fe_2_O_3_ nanoparticles. In addition, the hematite in the bulk form is antiferromagnetic with a weak ferromagnetism above the Morin transition and below its Néel temperature. However, the ferromagnetic behavior of the hematite at nano size has often been reported in literature due to the magnetic domain size which is reached at about 40 nm, but it is also affected by the morphology and crystal structure [38,39]. In the morphology analysis (Figure 3), a wide particle size distribution of α-Fe_2_O_3_ nanoparticles on the RGO surface can be seen, and also a large number of small nanoparticles (<10 nm), which are expected to have the monodomain size and exhibit ferromagnetic/superparamagnetic behavior dependent of the particle size [39]. According to the magnetic studies α-Fe_2_O_3_/RGO, nanocomposites exhibit a ferromagnetic behavior with small coercivity and remnant magnetization at room temperature, which is desirable for many practical applications, such as water purification systems, as it can be removed from the contaminated water. Figure 7 presents α-Fe_2_O_3_/RGO hybrid material dispersed in a pH 4 buffer solution placed beside RGO dispersion as control. The hybrid material was separated after being exposed to the magnetic field. However, additional studies should be done since α-Fe_2_O_3_ nanoparticles reduce the adsorption capability of RGO. The magnetization is probably enough to allow magnetic separation in laboratory-scale systems but might not be high enough to allow separation in large wastewater volumes.

## 4. Conclusions

Hybrid nanomaterials of RGO with α-Fe_2_O_3_ nanoparticles were prepared in situ by thermal treatment. The structural and morphology studies show that reduction of oxygen functional groups in RGO, after thermal treatment, and the presence of α-Fe_2_O_3_ nanoparticles on RGO surface impact the surface chemistry. The tetracycline adsorption on RGO depended on the pH value, and the maximum adsorption was 38.55 mg/g, 32.88 mg/g, and 14.58 mg/g at pH of 7, 10, and 4, respectively, in accordance with the Langmuir isotherm model. The adsorption kinetics fit well with the pseudo-second-order kinetic model. α-Fe_2_O_3_ nanoparticles grew on adsorption sites on RGO then the adsorption of tetracycline on the α-Fe_2_O_3_/RGO nanocomposite decreased. The α-Fe_2_O_3_/RGO nanocomposites exhibited a ferromagnetic behavior at room temperature that enabled the separation of their aqueous dispersions by using a magnetic field. However, additional studies should be done to use magnetic separation of this material in large scale wastewater treatment.

## Figures and Tables

**Table 1:** Summary of Tetracycline adsorption on carbon nanomaterials and composites.

Adsorbent	*Q_max_* (mg g^−1^) ^1^	Adsorption Conditions	Additional Functionality	Reference
GO	322.43	298 K; pH 3.6		[17]
GO	313.48	298 K; pH 3.6		[16]
CNT	269.54	293 K; pH 4.5–7		[15]
MWCNT	364.37	298 K		[27]
Hydroxyapatite/GO	16.16	293 K; pH 5–6	Photocatalytic oxidation	[28]
Fe_3_O_4_/GO	1272.45	313 K; pH 4	Magnetic removal	[29]
Fe/Cu/GO	302.5	pH 5–7	Magnetic removal	[20]
Fe_3_O_4_/GO sponge	473	308 K; pH 3	Magnetic removal	[30]

^1^*Q_max_* obtained from the fitting of adsorption isotherm with Langmuir model.

**Table 2:** Langmuir and Freundlich adsorption isotherms fitting parameters of tetracycline on α-Fe_2_O_3_/ RGO and RGO as control.

Adsorbent		Langmuir			Freundlich		
	pH	*Q_max_* (mg/g)	*K_L_* (L/mg)	r^2^	*n*	*K_F_* (L/mg)	r^2^
α-Fe_2_O_3_/RGO	4	18.48	0.30	0.965	2.71	5.79	0.971
	7	9.69	0.56	0.991	3.60	4.26	0.958
	10	10.25	0.35	0.945	3.77	4.20	0.894
RGO	4	15.82	0.05	0.921	1.44	0.99	0.993
	7	44.23	0.09	0.975	1.57	4.65	0.999
	10	39.94	0.07	0.994	1.31	2.83	0.995

## Supplementary Materials

The following are available online at https://www.mdpi.com/2079-4991/9/3/313/s1, Figure S1: FT-IR spectra of GO and RGO obtained after thermal treatment. Figure S2: XPS analysis of GO, RGO, FeCl_3_6H_2_O/GO, and α-Fe_2_O_3_/RGO hybrid material. (a) Spectra of O1s core and (b) Spectra Fe2p core. Figure S3: N2 adsorption-desorption isotherms for RGO and α-Fe_2_O_3_/RGO hybrid materials. Figure S4: Experimental curves of t/qt vs. t for the adsorption of TC on (a) RGO and (b) α-Fe_2_O_3_/RGO hybrid materials. Figure S5: Magnetic study of α-Fe_2_O_3_/RGO nanocomposites. (a) Temperature dependence of magnetization at a field of 40 kA/m and (b) hysteresis loop at room temperature for magnetic field up to 2 T. Table S1: Chemical composition from XPS analysis. Table S2: Parameters of pseudo-second-order kinetics for Tetracycline Adsorption on RGO and α-Fe_2_O_3_/RGO hybrid materials. Table S3: Experimental data of adsorption properties of tetracycline on α-Fe_2_O_3_/RGO hybrid materials: the amount of adsorbate adsorbed per unit weight of adsorbent (*q_e_*, mg/g) and the concentrations of adsorbate in the bulk solution (*C_e_*, mg/L) at a given temperature under equilibrium conditions.
