# Supplementary material for: Removal of Tetracycline Pollutants by Adsorption and Magnetic Separation Using Reduced Graphene Oxide Decorated with α-Fe2O3 Nanoparticles
Source: Nanomaterials (Basel). 2019 Feb 26;9(3):313. doi: 10.3390/nano9030313 (PMC6473670; doi:10.3390/nano9030313)
Supplement: Supplementary file 1 [file nanomaterials-09-00313-s001.pdf]

Supplementary Material

Removal of Tetracycline Pollutants by Adsorption and Magnetic Separation Using Reduced Graphene Oxide Decorated with α-Fe_2O_3 Nanoparticles

Adriana Magdalena Huízar-Félix [1], Celia Aguilar-Flores [1], Azael Martínez-de-la Cruz [1], José Manuel Barandiarán [2], Selene Sepúlveda-Guzmán [1,*] and Rodolfo Cruz-Silva [3]

[1] Universidad Autónoma de Nuevo León, UANL, Facultad de Ingeniería Mecánica y Eléctrica, FIME, Ave. Pedro de Alba s/n, Ciudad Universitaria, C.P. 66455, San Nicolás de los Garza, N.L., Mexico; adriana.mhuizarf@gmail.com (A.M.H.-F.); chemistry.aguilar@gmail.com (C.A.-F.); azael70@gmail.com (A.M.C.)

[2] Departamento de Electricidad y Electrónica, Universidad del País Vasco (UPV/EHU), 48940 Leioa, Spain; manub@we.lc.ehu.es

[3] Global Aqua Innovation Center and Institute of Carbon Science and Technology, Shinshu University 4-17-1 Wakasato, Nagano 380-8553, Japan; rcruzsilva.rcen@gmail.com

* Correspondence: selene.sepulvedagz@uanl.edu.mx; Tel.: +52-8183-29400

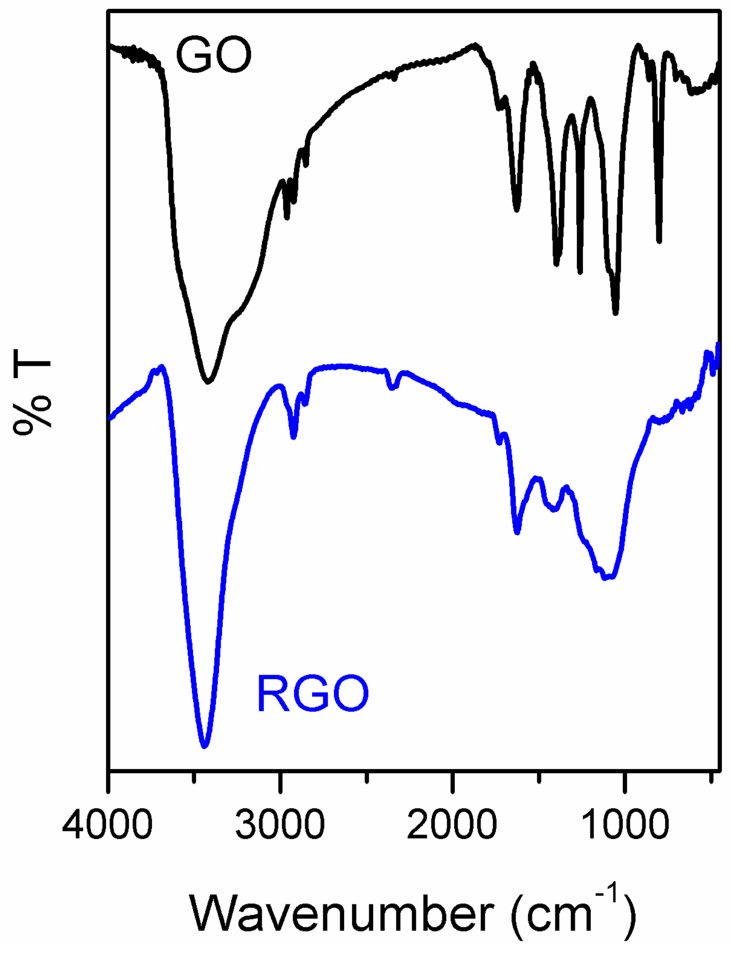

Figure 1. FT-IR spectra of GO and RGO obtained after thermal treatment.

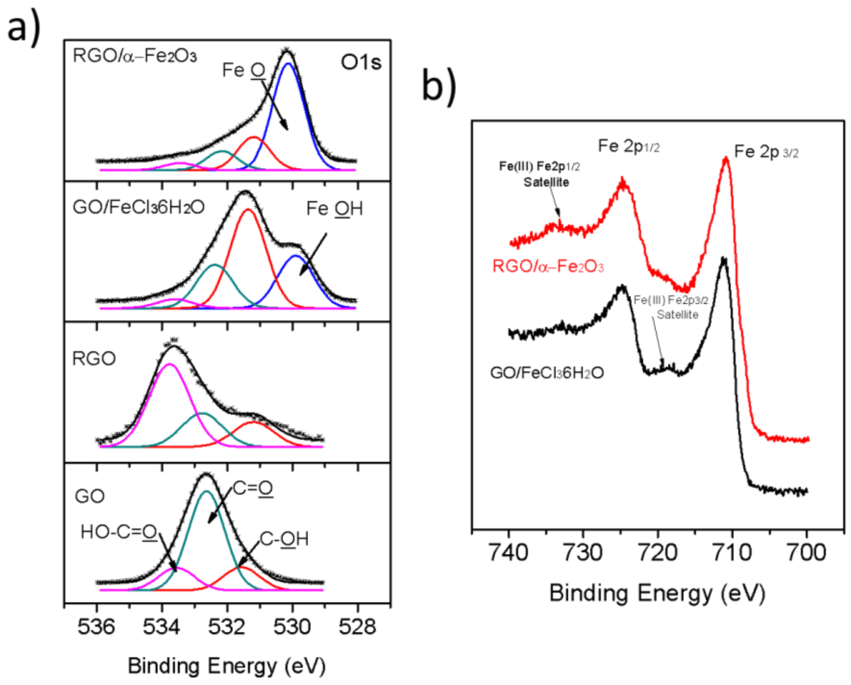

Figure 2. XPS analysis of GO, RGO, FeCl36H2O/GO and α-Fe_2O_3/RGO hybrid material. (**a**) Spectra of O1s core and (**b**) Spectra Fe2p core.

Table 1. Chemical composition from XPS analysis.

Sample	Surface area (m^2g^{-1})	Relative composition		C1s Peak Position (eV)						Atomic %					
		O/C	Fe/C	C-C (sp2)	C-C (sp3)	C-OH	C-O-C	C=O	COO	C-C (sp2)	C-C (sp3)	C-OH	C-O-C	C=O	COO
GO		0.40	-	284.0	284.9	285.9	286.9	287.9	289.0	6.0	29.5	8.5	44.0	7.6	4.5
RGO	327	0.07	-	284.0	284.8	285.8	286.6	287.7	288.9	5.8	61.8	15.0	9.4	4.9	3.1
FeCl36H2O/GO		0.53	0.11	284.0	284.8	285.9	286.8	288.0	289.0	7.8	63.1	12.4	6.9	4.1	5.7
α-Fe2O3/RGO	35	0.62	0.17	284.0	284.8	285.7	286.8	288.1	289.1	6.7	65.8	15.9	5.4	3.5	2.7

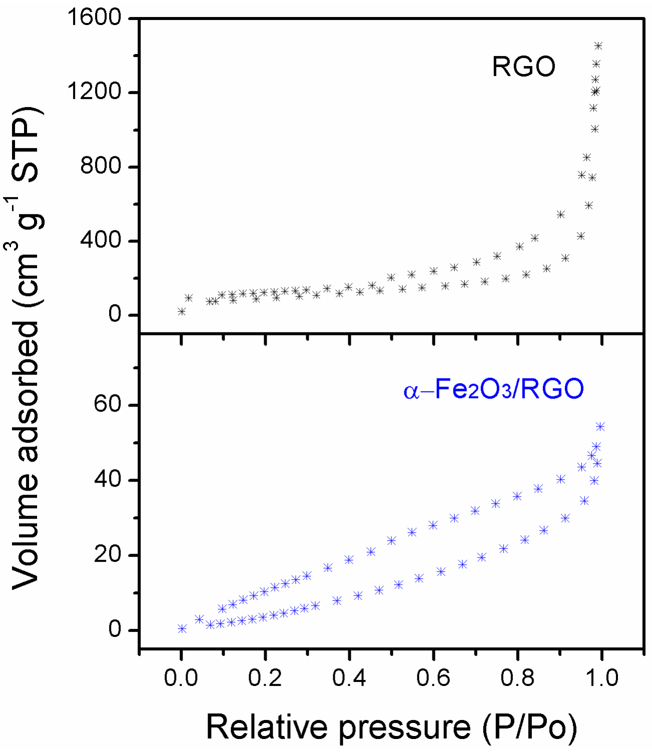

Figure 3. N_2 adsorption-desorption isotherms for RGO and α-Fe2O3/RGO hybrid materials.

The adsorption kinetics was modeled using the pseudosecond order model, which is expressed in Equation 1:

$$\frac{dq_t}{dt} = k_2(q_e - q_t)^2 \quad (1)$$

where q_e is the amount of solute adsorbed (mg/g) at equilibrium, q_t is the amount of solute adsorbed in time (min), k_2 is the rate constant of the pseudosecond order adsorption (mg/g·min). By integrating Equation 2 with the boundary conditions of qt = 0, t = 0 y qt = qt, t = t, the following linear equation is obtained:

$$\frac{t}{q_t} = \frac{k_2}{q_e^2} + \frac{1}{q_e t} \quad (2)$$

The q_e y k_2 values can be determined plotting t/q_t versus t. Figure S4 shows the curve of the experimental data by the pseudo-second order model, and the parameters for each system are presented in Table S2. The fits suggest that the tetracycline adsorption on RGO and α-Fe2O3/RGO hybrid materials obeys the Kinetic model of pseudo-second order.

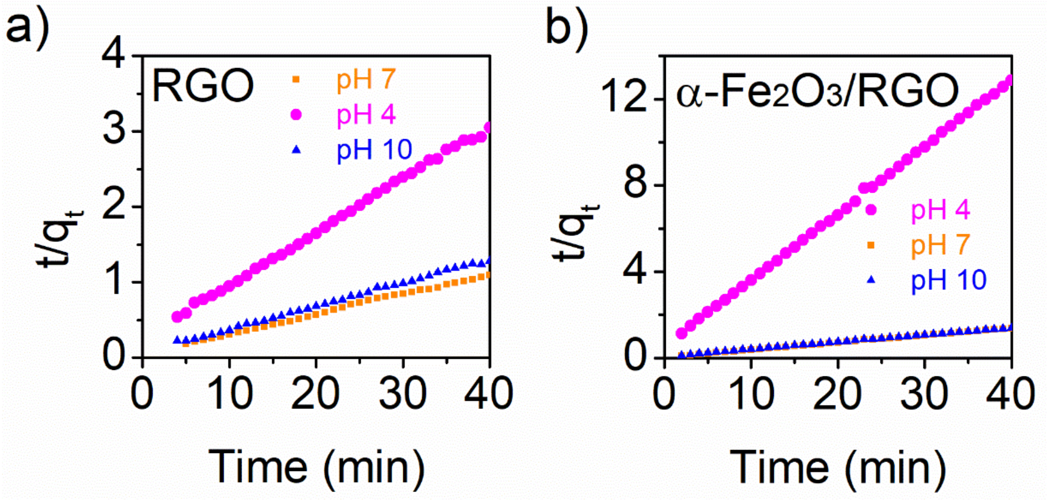

Figure 4. Experimental curves of t/q_t vs t for the adsorption of TC on (**a**) RGO and (**b**) α-Fe2O3/RGO hybrid materials.

Table 2. Parameters of Pseudo-second Order Kinetics for Tetracycline Adsorption on RGO and α-Fe_2O_3/RGO hybrid materials.

Adsorbent	pH	Pseudo-second-order kinetics		
		q_e (mg/g)	K_2 (g/mg·min)	r^2
α-Fe_2O_3/RGO	4	3.21	4.77	0.999
	7	29.33	23.67	0.999
	10	29.85	59.16	0.999
RGO	4	15.82	0.05	0.999
	7	44.23	0.09	0.999
	10	39.94	0.07	0.999

Table 3. Experimental data of adsorption properties of tetracycline on α-Fe_2O_3/RGO hybrid materials: amount of adsorbate adsorbed per unit weight of adsorbent (q_e, mg/g) and the concentrations of adsorbate in the bulk solution (C_e, mg/L) at a given temperature under equilibrium conditions.

pH 4		pH 7		pH 10	
C_e (mg/L)	q_e (mg/g)	C_e (mg/L)	q_e (mg/g)	C_e (mg/L)	q_e (mg/g)
13.01	15.87	16.75	8.51	13.75	9.39
10.63	14.06	11.19	9.31	11.00	7.98
7.32	10.52	8.31	7.55	8.09	6.06
3.02	9.48	4.74	6.71	4.30	6.58
1.28	6.23	1.69	4.79	1.67	4.87

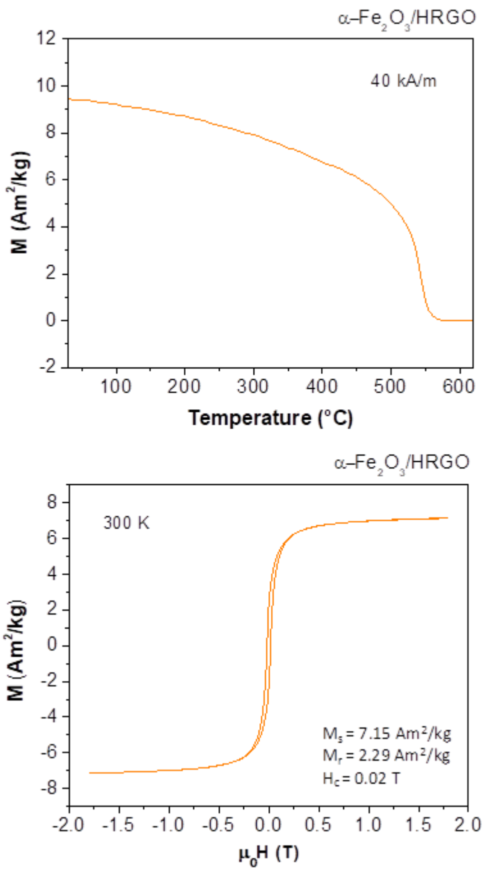

Figure 5. Magnetic study of α-Fe_2O_3/RGO nanocomposites. (**a**) Temperature dependence of magnetization at a field of 40 kA/m and (**b**) hysteresis loop at room temperature for magnetic field up to 2 T.
